# Surgical volume and outcomes of surgical ablation for atrial fibrillation: a nationwide population-based cohort study

**DOI:** 10.1186/s12872-023-03101-5

**Published:** 2023-02-11

**Authors:** Feng-Cheng Chang, Yu-Tung Huang, Victor Chien-Chia Wu, Hui-Tzu Tu, Chia-Pin Lin, Jih-Kai Yeh, Yu-Ting Cheng, Shang-Hung Chang, Pao-Hsien Chu, An-Hsun Chou, Shao-Wei Chen

**Affiliations:** 1 Department of Anesthesiology, Chang Gung Memorial Hospital, Linkou Medical Center, Chang Gung University, Taoyuan City, Taiwan; 2 Center for Big Data Analytics and Statistics, Chang Gung Memorial Hospital, Linkou Medical Center, Chang Gung University, Taoyuan City, Taiwan; 3 Department of Cardiology, Chang Gung Memorial Hospital, Linkou Medical Center, Chang Gung University, Taoyuan City, Taiwan; 4 Division of Thoracic and Cardiovascular Surgery, Department of Surgery, Chang Gung Memorial Hospital, Linkou Medical Center, Chang Gung University, No. 5, Fuxing St., Guishan Dist., Taoyuan City, 33305 Taiwan

**Keywords:** Atrial fibrillation, Surgical ablation, Volume-outcome, Maze procedures, Survival analysis

## Abstract

**Background:**

Atrial fibrillation is the most common cardiac arrythmia and causes many complications. Sinus rhythm restoration could reduce late mortality of atrial fibrillation patients. The Maze procedure is the gold standard for surgical ablation of atrial fibrillation. Higher surgical volume has been documented with favorable outcomes of various cardiac procedures such as mitral valve surgery and aortic valve replacement. We aimed to determine the volume–outcome relationship (i.e., association between surgical volume and outcomes) for the concomitant Maze procedure during major cardiac surgeries.

**Methods:**

This nationwide population-based cohort study retrieved data from the Taiwan National Health Insurance Research Database. Adult patients undergoing concomitant Maze procedures during 2010–2017 were identified; consequently, 2666 patients were classified into four subgroups based on hospital cumulative surgery volumes. In-hospital outcomes and late outcomes during follow-up were analyzed. Logistic regression and Cox proportional hazards model were used to analyze the volume–outcome relationship.

**Results:**

Patients undergoing Maze procedures at lower-volume hospitals tended to be frailer and had higher comorbidity scores. Patients in the highest-volume hospitals had a lower risk of in-hospital mortality than those in the lowest-volume hospitals [adjusted odds ratio, 0.30; 95% confidence interval (CI), 0.15–0.61; *P* < 0.001]. Patients in the highest-volume hospitals had lower rates of late mortality than those in the lowest-volume hospitals, including all-cause mortality [adjusted hazard ratio (aHR) 0.53; 95% CI 0.40–0.68; P < 0.001] and all-cause mortality after discharge (aHR 0.60; 95% CI 0.44–0.80; P < 0.001).

**Conclusions:**

A positive hospital volume–outcome relationship for concomitant Maze procedures was demonstrated for in-hospital and late follow-up mortality. The consequence may be attributed to physician skill/experience, experienced multidisciplinary teams, and comprehensive care processes. We suggest referring patients with frailty or those requiring complicated cardiac surgeries to high-volume hospitals to improve clinical outcomes.

*Trial registration*: the institutional review board of Chang Gung Memorial Hospital approved all data usage and the study protocol (registration number: 202100151B0C502).

**Supplementary Information:**

The online version contains supplementary material available at 10.1186/s12872-023-03101-5.

## Background

Atrial fibrillation (AF) is the most common cardiac arrythmia and causes many complications, including stroke, heart failure, and dementia [1]. The prevalence of AF in the United States is expected to reach 5.6–15.9 million by 2050 [1, 2]. Globally, approximately 5 million new cases are identified annually. AF significantly increases the burden of healthcare and medical expenses. In Taiwan, the prevalence of AF is about 1.1% (1.4% in men and 0.7% in women), lower than 2% in the white population. The prevalence of AF increased with advanced ages in Taiwanese population and the annual frequency of hospitalization for AF increased during the past decade [3]. This trend in Taiwan was concordant with the United States. Moreover, one previous cohort study reported the average in-hospital mortality rate was 9.3% in Taiwan compared to 1% in the United States [4, 5]. To maintain sinus rhythm and further restore myocardial function, pharmacologic and nonpharmacological strategies are used to treat AF in clinical practice.

Surgical ablation is currently the mainstream strategy for treating or preventing AF. The Maze procedure is the most commonly performed surgical ablation procedure and is the gold standard [6]. This technique is usually concomitantly performed with major cardiac surgery (coronary bypass grafting, valve surgery, or aortic surgery) [7]. One previous meta-analysis demonstrated 12 months of freedom from AF after concomitant surgical ablation [8]. Although major cardiac surgeries have unfavorable outcomes if AF is left untreated [6, 7, 9, 10], McCarthy et al. demonstrated that only 22.1% of AF patients undergoing cardiac surgeries underwent concurrent surgical ablation [7]. One recent study was conducted to investigate the long-term outcomes of concomitant surgical ablation for atrial fibrillation in Taiwan. Cheng et al. reported the concomitant AF ablation is safe during various types of cardiac procedures with favorable survival outcome than general AF population [11].

Surgeons’ experience may affect the outcomes of the concomitant Maze procedure [10, 12, 13]. Volume–outcome relationships (i.e., associations between the surgical volume and outcomes) for many cardiac procedures have been reported in previous studies [14–16]. Christina et al. reported that patients in lower-volume hospitals demonstrated higher operative mortality following mitral valve surgery and lower repair rates [15].In addition, Himanshu et al. demonstrated a positive volume–outcome relationship for aortic valve replacement [14]. However, the effects of procedural volume on Maze procedure outcomes are not well established. The population-based claims database in Taiwan provides valuable data as it is a large-scale database with universal coverage [17]. The present study aimed to determine the relationship between the surgical volume for the Maze procedure and its short- and long-term outcomes and to assess patient and hospital characteristics in different volume subgroups.

## Methods

### Data source

This population-based cohort study was conducted using data from the Taiwan National Health Insurance Research Database (NHIRD). The NHIRD originated from the National Health Insurance (NHI) program in Taiwan, which covers approximately 99.8% of Taiwan residents and is a government-operated singer-payer system. The NHI has reimbursed universal medical expenditures for hospitalization healthcare, outpatient visits, major surgeries and associated medical treatments since 1995 [18]. Therefore, the NHIRD provides detailed data for patients who underwent major cardiac surgeries with concomitant surgical ablation, including cardiac procedures, demographic distribution, underlying comorbidities, and mortality and readmission outcomes. Consequently, this is a practical and valid source of data for the present study. All patient data are de-identified (anonymized) and only on-site analyses at the Health and Welfare Data Center established by the Ministry of Health and Welfare are allowed, due to privacy concerns and patient protection. This study was reviewed and approved via the NHIRD research committee and the institutional review board of Chang Gung Memorial Hospital (registration number: 202100151B0C502). The need for individual informed consent was waived.

### Study population

**Fig. 1:**
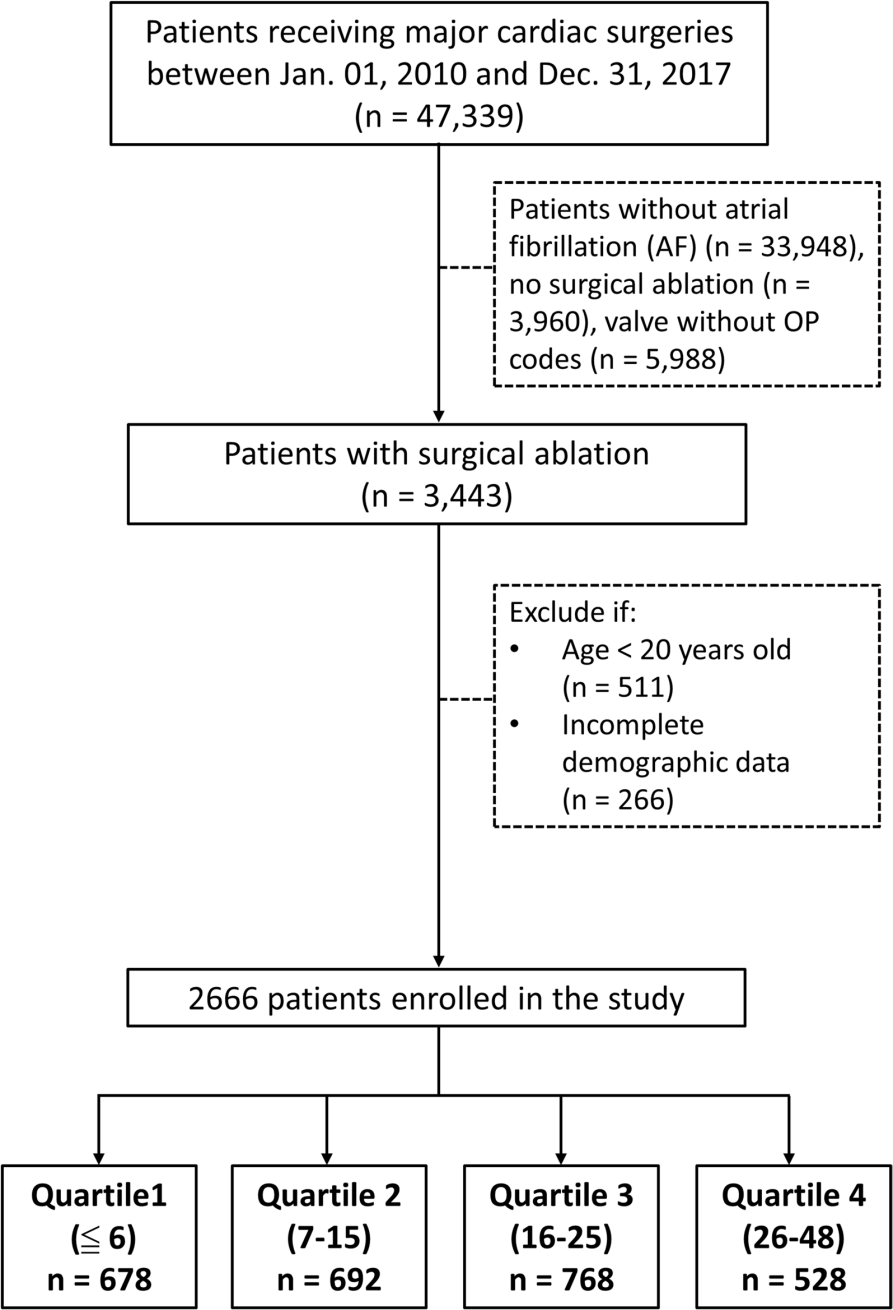
Flowchart for the inclusion of study patients

We identified major cardiac surgeries and concomitant Maze procedures using the NHI reimbursement codes, according to the International Classification of Disease (ICD), Ninth Revision, Clinical Modification (ICD-9-CM) procedure codes (before December 31, 2015), and the Tenth Revision (ICD-10-CM) procedure codes (after January 1, 2016) (Additional file 1: Table S1). Patients (≥ 20 years), who underwent major cardiac surgeries, were initially identified using NHI reimbursement codes and ICD procedure codes between January 1, 2010, and December 31, 2017. Patients without demographic data, precise cardiac procedure codes, diagnosis of AF, or surgical ablation were excluded to ensure the adequacy of enrollment. After applying the inclusion and exclusion criteria, 2666 patients were eligible for analysis (Fig. 1).

**Table 1:** Demographic and surgical characteristics of the patients according to the quartile of cumulative hospital volume

	Total		Quartile 1 (≦6)		Quartile 2 (7–15)		Quartile 3 (16–25)		Quartile 4 (26–48)		P value
	n	%	N	%	n	%	n	%	n	%	
Subjects	2666	100	678	25.4	692	26.0	768	28.8	528	19.8	–
Age (years), mean ± SD	63	11	64	11	65	11	61	12	64	11	< 0.001
Male	1382	51.8	350	51.6	363	52.5	394	51.3	275	52.1	0.97
Urbanization level of the residence											< 0.001
Urban	1553	58.2	352	51.9	356	51.5	479	62.4	366	69.3	
Suburban	832	31.2	213	31.4	247	35.7	237	30.9	135	25.6	
Rural	281	10.5	113	16.67	89	12.9	52	6.8	27	5.1	
Hospital level											< 0.001
Medical centers	700	65.4	291	42.9	692	100.0	768	100.0	528	100.0	
Regional hospitals	379	34.9	379	55.9	0	0.0	0	0.0	0	0.0	
District hospitals	8	0.3	8	1.2	0	0.0	0	0.0	0	0.0	
Comorbid conditions											
Diabetes mellitus	594	22.3	159	23.5	174	25.1	146	19.0	115	21.8	0.035
Hypertension	1348	50.6	352	51.9	386	55.8	360	46.9	250	47.4	0.002
Dyslipidemia	605	22.7	162	23.9	142	20.5	156	20.3	145	27.5	0.008
CKD	502	18.8	150	22.1	140	20.2	130	16.9	82	15.5	0.010
Rheumatic heart disease	1331	49.9	333	49.1	333	48.1	412	53.7	253	47.9	0.10
Malignant dysrhythmia	43	1.6	13	1.9	4	0.6	11	1.4	15	2.8	0.017
Infective endocarditis	55	2.1	15	2.2	15	2.2	19	2.5	6	1.1	0.39
Myocardial infarction	118	4.4	36	5.3	30	4.3	36	4.7	16	3.0	0.28
History of event											
History of PCI	23	0.9	7	1.0	16	2.3	4	0.5	1	0.2	< 0.001
History of Heart failure	1170	43.9	324	47.8	313	45.2	313	40.8	220	41.7	0.032
Prior stroke	242	9.1	82	12.1	66	9.5	53	6.9	41	7.8	0.004
History of GI bleeding	253	9.5	74	10.9	70	10.1	58	7.6	51	9.7	0.15
Previous cardiac surgery	6	0.2	0	0.0	3	0.4	2	0.3	1	0.2	0.39
Emergency surgery	1	0.04	1	0.2	0	0.0	0	0.0	0	0.0	–
Charlson’s Comorbidity Index total score, mean ± SD	2.22	1.72	2.44	1.84	2.37	1.81	2.06	1.57	1.96	1.58	< 0.001
CHA_2_DS_2_–VAS_C_ score, mean ± SD	2.94	1.80	3.07	1.83	3.17	1.86	2.62	1.73	2.95	1.71	< 0.001
HAS-BLED score, mean ± SD	1.78	1.28	1.90	1.32	1.91	1.31	1.58	1.24	1.75	1.28	< 0.001
Type of cardiac surgery											
CABG	302	11.3	83	12.2	58	8.4	67	8.7	94	17.8	< 0.001
Mechanical AVR	184	6.9	71	10.5	38	5.5	37	4.8	38	7.2	< 0.001
Tissue AVR	406	15.2	75	11.1	135	19.5	119	15.5	77	14.6	< 0.001
MV repair	748	28.1	129	19.0	96	13.9	367	47.8	156	29.6	< 0.001
Mechanical MVR	603	22.6	234	34.5	137	19.8	133	17.3	99	18.8	< 0.001
Tissue MVR	970	36.4	222	32.7	361	52.2	174	22.7	213	40.3	< 0.001
TV repair	649	24.3	188	27.7	128	18.5	216	28.1	117	22.2	< 0.001
TVR	59	2.2	12	1.8	29	4.2	13	1.7	5	1.0	< 0.001
Aorta surgery	32	1.2	13	1.9	3	0.4	12	1.6	4	0.8	0.044
Follow-up (years), mean ± SD	2.94	2.31	2.68	2.34	2.69	2.16	3.16	2.32	3.25	2.35	< 0.001

Quartile 1, lowest; Quartile 4, highest; SD, standard deviation; CKD, chronic kidney disease; PCI, percutaneous coronary intervention; GI, gastrointestinal; CABG, coronary artery bypass graft; AVR, aortic valve replacement; MV, mitral valve; MVR, mitral valve replacement; TV, tricuspid valve; TVR, tricuspid valve replacement. HAS-BLED score is calculated by assigning a score of 1 point for each of the following conditions: hypertension, abnormal liver/renal function, stroke, bleeding, age, drug used(NSAIDs, Aspirin, Clopidogrel), alcoholism

The CHA2DS2-VASc risk score is calculated by assigning a score of 1 point for each of the following conditions: congestive heart failure (ejection fraction < 40%), hypertension, age between 65 and 74 years, diabetes mellitus, vascular disease (myocardial infarction or peripheral arterial disease), and female gender; and a score of 2 points for the following conditions: history of stroke or transient ischemic attack (TIA) and age > 75 years

### Cumulative hospital volume of Maze procedures

All hospitals had distinctive codes and were deidentified. Annual hospital volumes were defined by the mean number of Maze procedures per year [19]. Initially, we calculated the annual procedure volume of all hospitals during the study period (2010–2017). The hospitals were ranked sequentially according to the annual procedure volume. The cumulative hospital volumes during the study period were then calculated for all hospitals. The hospitals were further divided into four subgroups with comparable cumulative surgical volumes (quartiles) [19]. Consequently, all four subgroups comprised approximately 25% of the total cumulative surgery volume and were categorized as lowest (N = 678; annual volume ≤ 6; Q1), low–moderate (N = 692; annual volume: 7–15; Q2), moderate–high (N = 768; annual volume: 16–25; Q3), and highest-volume (N = 528; annual volume 26–48; Q4) hospitals. This classification was also conducted in our previous study [20].

### Covariates

We analyzed the following covariates: age, sex, the level of urbanization of the patient’s residence (urban, suburban, or rural), hospital level (medical center, regional hospital or district hospital), comorbidities (diabetes mellitus, hypertension, dyslipidemia, chronic renal insufficiency, rheumatic heart disease, malignant dysrhythmia, infective endocarditis, myocardial infarction, heart failure, stroke, gastrointestinal bleeding, and prior percutaneous coronary intervention), presence of surgery urgency, Charlson’s Comorbidity Index score, CHA_2_DS_2_-VASc score [21], HAS-BLED score [22], and the type of major cardiac surgery (see Table 1 for the detailed descriptions of the covariates). The comorbidities were detected using ICD diagnostic codes (Additional file 1: Table S1).

### Outcomes

In-hospital outcomes and late outcomes during follow-up were analyzed. The outcomes of primary interest were in-hospital mortality and all-cause mortality after discharge (Table 2). The secondary outcomes were in-hospital perioperative complications (Table 3) and follow-up outcomes, including readmission and major adverse cardiovascular events (MACE). In-hospital mortality was defined as death during index hospitalization. MACE involved acute myocardial infarction, stroke, and cardiovascular mortality during follow-up. Mortality was determined using the Death Registry dataset with anonymized identification number.

**Table 2:** Long-term outcomes during the follow-up period

	Events		Unadjusted model		Adjusted model*	
	N	%	HR, SHR (95%CI)	P value	HR, SHR (95%CI)	P value
All-cause mortality^¶^						
Quartile 1	194	28.6	Reference		Reference	
Quartile 2	188	27.2	0.95 (0.77–1.16)	0.59	0.99 (0.80–1.22)	0.90
Quartile 3	133	17.3	0.53 (0.43–0.66)	< 0.001	0.72 (0.57–0.91)	0.006
Quartile 4	84	15.9	0.48 (0.37–0.62)	< 0.001	0.53 (0.40–0.68)	< 0.001
P for linear trend	–	–	–	< 0.001	–	< 0.001
All-cause mortality after discharge^¶^						
Quartile 1	140	22.4	Reference		Reference	
Quartile 2	147	22.6	1.03 (0.82–1.30)	0.78	1.08 (0.84–1.37)	0.55
Quartile 3	106	14.3	0.57 (0.44–0.74)	< 0.001	0.71 (0.54–0.93)	0.012
Quartile 4	73	14.1	0.56 (0.42–0.74)	< 0.001	0.60 (0.44–0.80)	< 0.001
P for linear trend	–	–	–	< 0.001	–	< 0.001
Readmission [CV-related causes]^§^						
Quartile 1	333	53.4	Reference		Reference	
Quartile 2	332	51.0	0.90 (0.77–1.05)	0.19	0.94 (0.80–1.11)	0.46
Quartile 3	336	45.3	0.71 (0.61–0.83)	< 0.001	0.81 (0.69–0.94)	0.008
Quartile 4	265	51.3	0.89 (0.76–1.05)	0.16	0.93 (0.78–1.10)	0.37
Readmission [all cause, within 30 days]^§^						
Quartile 1	79	12.7	Reference		Reference	
Quartile 2	68	10.5	0.82 (0.59–1.13)	0.097	0.88 (0.63–1.23)	0.45
Quartile 3	56	7.6	0.57 (0.40–0.80)	0.001	0.67 (0.46–0.97)	0.03
Quartile 4	53	10.3	0.75 (0.53–1.05)	0.21	0.81 (0.57–1.16)	0.25
MACE^¶^						
Quartile 1	45	7.2	Reference		Reference	
Quartile 2	57	8.8	1.25 (0.85–1.85)	0.26	1.39 (0.93–2.09)	0.11
Quartile 3	57	7.7	1.03 (0.70–1.52)	0.87	1.23 (0.80–1.87)	0.35
Quartile 4	40	7.7	1.05 (0.69–1.60)	0.83	1.17 (0.75–1.81)	0.49
Oral anticoagulation therapy^§^						
Quartile 1	207	33.2	Reference		Reference	
Quartile 2	229	35.2	1.04 (0.86–1.26)	0.68	1.25 (1.02–1.53)	0.029
Quartile 3	286	38.6	1.10 (0.92–1.32)	0.28	1.44 (1.20–1.74)	< 0.001
Quartile 4	97	18.8	0.50 (0.39–0.63)	< 0.001	0.56 (0.44–0.72)	< 0.001

Quartile 1, lowest; Quartile 4, highest. The event numbers and rates for each quartile are expressed as total number (N) and proportions (%), respectively

CI, confidence interval; MACE, major adverse cardiovascular events

^¶^HR, hazard ratio; ^§^SHR, sub-distribution hazard ratio

^¶^The risk of fatal outcomes for volume groups were analyzed using Cox proportional hazard model

^§^The nonfatal outcomes for volume groups were analyzed using competing risk analysis

*All adjustment variables are presented in Table 1

**Table 3:** In-hospital outcomes and operation-related complications according to the quartile of cumulative hospital volume^#^

	Events		Unadjusted model		Adjusted model*	
	N	%	OR (95%CI)	P value	OR (95%CI)	P value
In-hospital mortality						
Quartile 1	54	8.0	Reference		Reference	
Quartile 2	41	5.9	0.73 (0.48–1.11)	0.14	0.75 (0.47–1.19)	0.22
Quartile 3	27	3.5	0.42 (0.26–0.68)	< 0.001	0.74 (0.44–1.25)	0.26
Quartile 4	11	2.1	0.25 (0.13–0.48)	< 0.001	0.30 (0.15–0.61)	< 0.001
Cardiogenic shock requiring mechanical circulatory support						
Quartile 1	67	9.9	Reference		Reference	
Quartile 2	58	8.4	0.83 (0.58–1.21)	0.34	0.87 (0.58–1.29)	0.49
Quartile 3	39	5.1	0.49 (0.32–0.73)	< 0.001	0.61 (0.39–0.95)	0.029
Quartile 4	17	3.2	0.31 (0.18–0.52)	< 0.001	0.35 (0.20–0.61)	< 0.001
Re-exploration for bleeding						
Quartile 1	17	2.5	Reference		Reference	
Quartile 2	22	3.2	1.28 (0.67–2.43)	0.46	1.43 (0.73–2.81)	0.29
Quartile 3	24	3.1	1.25 (0.67–2.36)	0.48	1.37 (0.70–2.70)	0.36
Quartile 4	0	0.00	–	–	–	–
De novo dialysis						
Quartile 1	110	16.2	Reference		Reference	
Quartile 2	61	8.8	0.50 (0.36–0.70)	< 0.001	0.48 (0.33–0.69)	< 0.001
Quartile 3	38	5.0	0.27 (0.18–0.40)	< 0.001	0.34 (0.22–0.51)	< 0.001
Quartile 4	24	4.6	0.25 (0.16–0.39)	< 0.001	0.29 (0.18–0.47)	< 0.001
Massive blood transfusion						
Quartile 1	28	5.1	Reference		Reference	
Quartile 2	69	11.9	2.54 (1.61–4.00)	< 0.001	2.78 (1.71–4.55)	< 0.001
Quartile 3	33	5.7	1.15 (0.68–1.92)	0.61	1.51 (0.87–2.63)	0.14
Quartile 4	11	3.0	0.59 (0.29–1.20)	0.14	0.64 (0.31–1.33)	0.24
Deep wound infection						
Quartile 1	36	5.3	Reference		Reference	
Quartile 2	24	3.8	0.64 (0.38–1.09)	0.10	0.58 (0.33–1.03)	0.063
Quartile 3	22	2.9	0.53 (0.31–0.90)	0.02	0.75 (0.42–1.35)	0.33
Quartile 4	7	1.3	0.24 (0.11–0.54)	< 0.001	0.28 (0.12–0.65)	0.003

Quartile 1, lowest; Quartile 4, highest. The event numbers and rates in each quartile were expressed as total number (N) and proportions (%), respectively. OR, odds ratio; CI, confidence interval. ^#^All outcomes were analyzed using logistic regression

^*^All adjustment variables are presented in Table 1

### Statistical analysis

The inter-volume (the quartiles of volume) patient characteristics (Table 1), were compared with one-way analysis of variance (ANOVA) and chi-squared tests. The data not normally distributed in Table 1 were compared by using the Kruskal–Wallis test. Logistic regression analyses were used to compare in-hospital mortality and associated complications between the quartiles of volume. For the late outcomes, the Cox proportional hazard model was used to compare inter-volume fatal outcomes including all-cause mortality, all-cause mortality after discharge, and MACE. The nonfatal outcomes were analyzed with the Fine and Gray sub-distribution hazard model, considering all-cause mortality as a competing risk. Patients with a presence of competing risk (e.g., mortality) remain to be followed for a while in the Fine and Gray model and therefore the estimated incidence of event would be lowered compared to other methods (e.g., Kaplan–Meier) [23]. Since the Taiwan national health insurance is compulsory and statutory, patients were hardly lost to follow-up. When patients were dead, the National Death Registry system recorded the causes of death. In this study, the index date we began to follow the patients were defined as the admission date of the index hospitalization. Thus, all patients across the study period were followed from the admission date of the index hospitalization until December 31, 2017 or the date of death, whichever came first. These regression models that were adjusted for covariates are listed in Table 1. The proportional hazard assumptions were tested by calculating Schoenfeld partial residuals. To assess the potential impact of hospital effects, we conducted a sensitivity analysis to incorporate cluster-specific random effects by treating hospitals as a cluster variable in a frailty survival model with gamma distribution [24]. The trend of distribution was tested using Joinpoint regression. A two-sided *P* value < 0.05 was considered statistically significant. Statistical analyses were performed using SAS version 9.4 (SAS Institute, Cary, NC) and Joinpoint Trend Analysis software version 4.7 (National Cancer Institute, Bethesda, MD).

## Results

### Distribution of patients in each quartile of the cumulative hospital volume

**Fig. 2:**
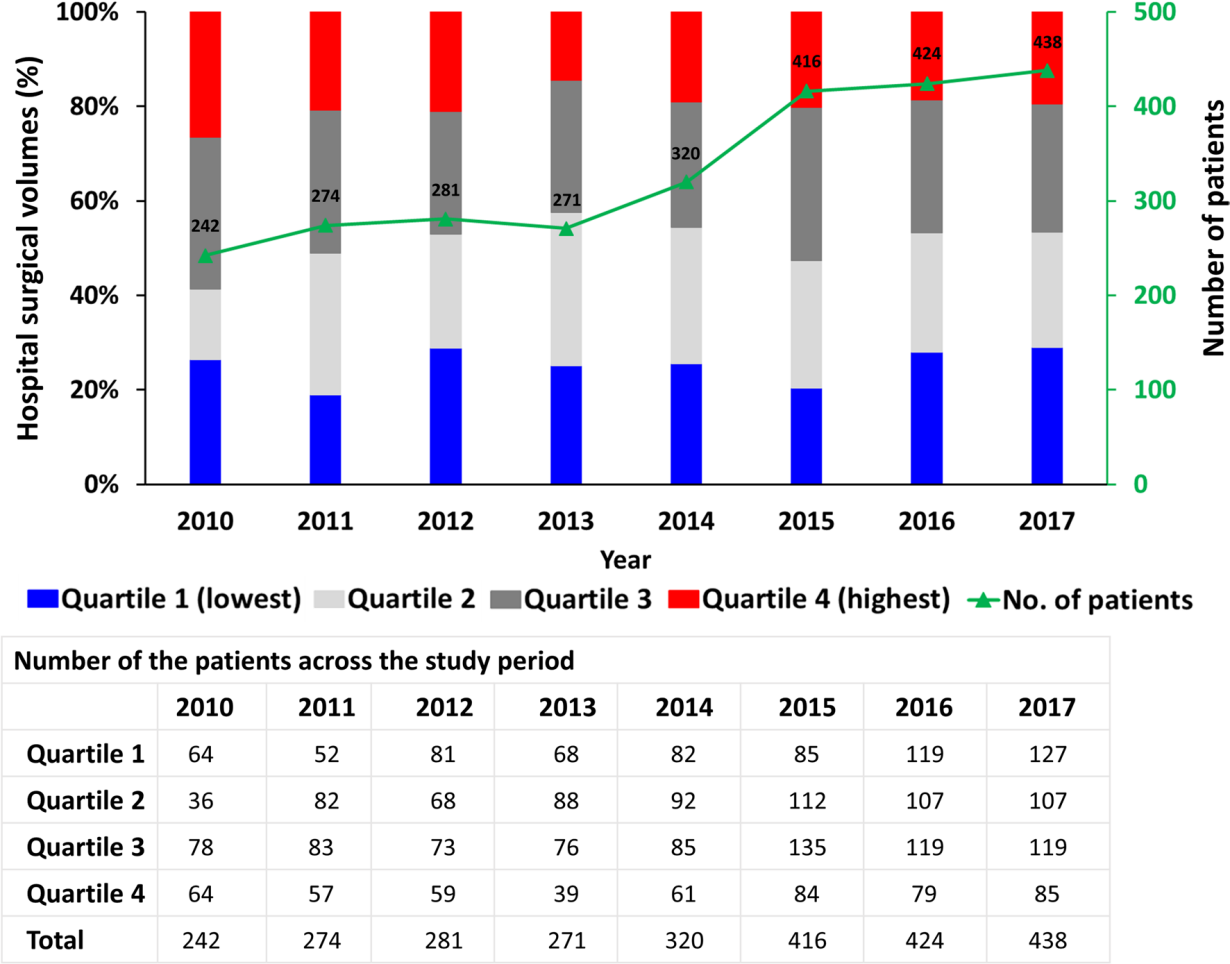
Distribution of patients in each quartile of cumulative hospital volume and trends of patients receiving surgical ablation during the study period. The total numbers of patients undergoing concomitant surgical ablation in each year are shown in green. The numbers of patients undergoing concomitant surgical ablation in each quartile during the study period are also shown

This study included 2666 patients undergoing concomitant Maze procedures from 2010 to 2017. Figure 2 shows the distribution of patients in each subgroup of cumulative hospital volumes during analysis. The number of patients increased from 242 in 2010 to 438 in 2017. No changes were observed in the proportion of procedures performed in each quartile during the observation period, with trend analysis indicating no statistically significant difference (Q1, P = 0.4344; Q2, P = 0.9916; Q3, P = 0.4799 and Q4, P = 0.16).

### Baseline characteristics

Table 1 shows the patient demographics and surgical characteristics among the four subgroups. The mean age of the entire cohort was 63 years; 51.8% of the patients were men, and the mean follow-up was 2.94 ± 2.31 years (mean ± standard deviation). Several comorbidity scores were more severe in patients from the Q1 subgroup including CHA2DS2-VASc, HAS-BLED, and Charlson Comorbidity Index. Coronary artery bypass surgery, mitral repair, and aortic and mitral valve replacements with tissue prostheses were more common concomitant major cardiac surgeries in the highest-volume hospitals (Q4) compared with concomitant surgeries in the Q1 hospitals. The rate of surgical ablation during cardiac surgeries in AF patients was 18.8% in the lowest-volume hospitals (Q1) and 47.4% in the highest-volume hospitals (Q4), respectively (Additional file 1: Table S2). In general, a higher surgical ablation rate was also observed in the Q4 hospitals for most types of cardiac procedures (Additional file 1: Table S3).

### Outcomes of primary interest

**Fig. 3:**
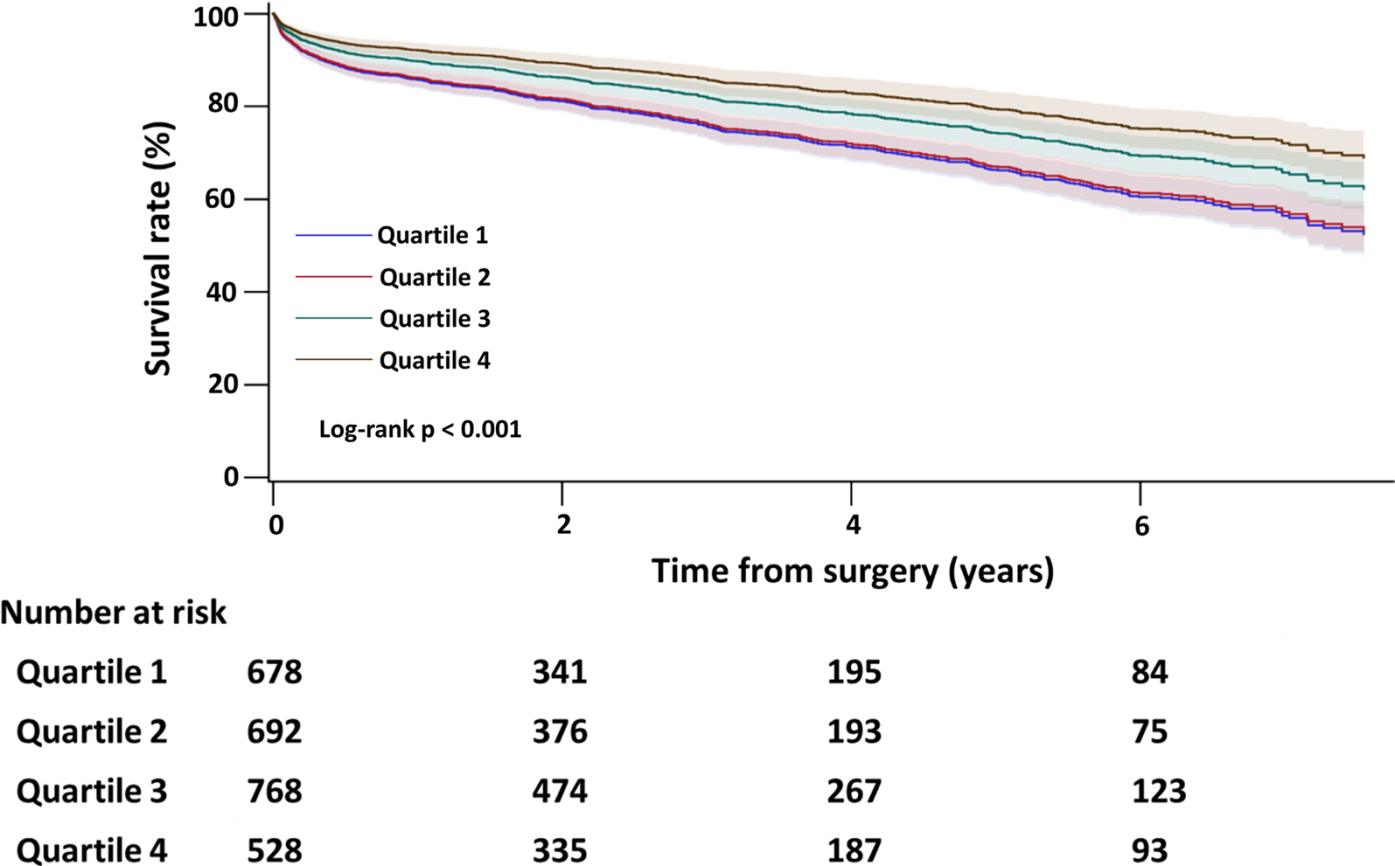
Kaplan–Meier survival curves demonstrating overall all-cause mortality for the four quartiles of cumulative hospital volumes

**Fig. 4:**
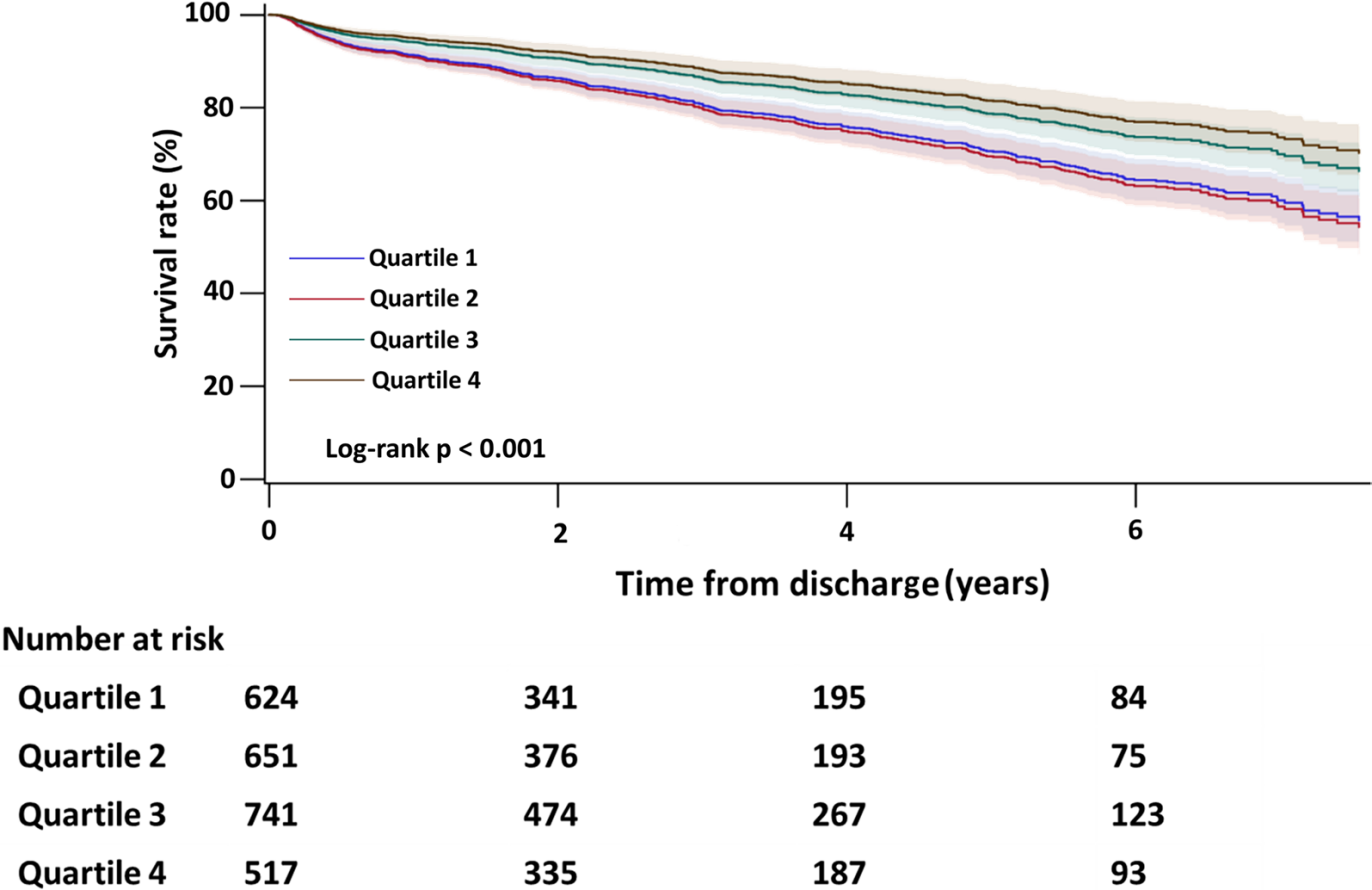
Kaplan–Meier survival curves demonstrating all-cause mortality after discharge for the four quartiles of cumulative hospital volumes

The all-cause mortality rates (Fig. 3) were generally higher in the lower-volume hospitals. Patients undergoing Maze procedures in the highest-volume hospitals had a lower risk of all-cause mortality than patients in the lowest-volume hospitals (HR 0.53; 95% CI 0.40–0.68; *P* < 0.001) (Table 2). The all-cause mortality after discharge (Fig. 4) showed similar results, with a lower mortality rate in the highest-volume hospitals compared with outcomes in the lowest-volume hospitals (HR, 0.60; 95% CI 0.44–0.80; *P* < 0.001). The linear trends tested for the all-cause mortality and discharge mortality during follow-up revealed statistical significance in both unadjusted and adjusted model (*P* for trend < 0.001), demonstrating the decreasing trend of mortality with increasing hospital volume. After covariate adjustment, patients undergoing Maze procedures in Q4 hospitals had a lower risk of in-hospital mortality than patients in the Q1 hospitals (adjusted odds ratio [OR], 0.30; 95% confidence interval [CI], 0.15–0.61; *P* < 0.001). In terms of the overall in-hospital mortality in this cohort, 133 (5.0%) patients died during the index admission. The overall real-world in-hospital mortality rate was higher in lower-volume hospitals (Q1: 8.0% vs. Q4: 2.1%; *P* < 0.001) (Table 3).

### Operation-related complications and follow-up outcomes

Patients in Q4 hospitals had a lower risk of postoperative cardiogenic shock requiring mechanical circulatory support (*P* < 0.001), de novo dialysis (*P* < 0.001), re-exploration for bleeding than those in the lower-volume hospitals. Table 2 shows the follow-up outcomes after covariate adjustments. The calculated Schoenfeld partial residuals for each outcome revealed no violation of the proportional hazard assumptions (Additional file 1: Table S4). Compared with the lowest-volume hospitals (Q1), we observed a lower rate of anticoagulation use in the highest-volume hospitals (Q4) (SHR 0.56; 95% CI 0.44–0.72; P < 0.001). Other late outcomes were relatively comparable between the four quartiles, including readmissions within 30 days, readmissions for cardiovascular causes, and MACE. The results of the sensitivity test (Additional file 1: Table S5) were consistent with the primary analysis in Table 2.

## Discussion

### Main findings

The present study demonstrates that the number of patients undergoing concomitant Maze procedures during major cardiac surgeries has increased in the last decade in Taiwan. Furthermore, surgical volume is related to short- and long-term outcomes. Lower-volume hospitals generally had less favorable short-term outcomes, including in-hospital mortality and operation-related complications, compared to higher-volume hospitals. In addition, the higher-volume hospitals had lower risks of late mortality. These results indicate a positive volume–outcome relationship (i.e., higher hospital surgical volume associated with more favorable outcomes) for the Maze procedure.

### Trends for Maze procedures performed in Taiwan

Aging and westernization trends in Taiwanese society predispose the population to AF risk factors, such as hypertension, coronary artery disease, ischemic heart disease, and degenerative valvular disease [25]. Therefore, the number of people undergoing cardiac procedures has increased steadily over the past decades. In Taiwan, the proportion of concomitant surgical ablation for AF patients during mitral repair, bioprosthetic mitral valve replacement (MVR), and mechanical MVR were 51.8%, 48.2%, and 39.1%, respectively. Furthermore, the surgical ablation rates during bioprosthetic aortic valve replacement (AVR), mechanical AVR, and coronary bypass surgery were 29.4%, 24.4%, and 13.5%, respectively (Additional file 1: Table S3). McCarthy et al. reported similar results; 37.6% of AF patients undergoing mitral valve surgery also underwent surgical ablation, while only 16.4% of patients undergoing non-MV procedures [7]. Notably, patients undergoing non-MV procedures had lower rates of concomitant surgical ablation. Surgeons may be inclined to not perform surgical ablation when opening of the left atrium is not required and surgical ablation would be more technically challenging [7]. Moreover, additional surgical ablation may prolong the surgery duration and bypass time, contributing to worse outcomes. This condition was also observed in our cohort, the rate of concurrent surgical ablation for AF patients during cardiac surgeries was higher in the higher-volume hospitals (47.4% in Q4 and 18.8% in Q1, respectively). Nevertheless, untreated AF in patients undergoing major surgery results in unfavorable outcomes [6, 9, 10, 26]. Many previous studies demonstrated that concomitant Maze procedures in aortic valve surgery or coronary bypass surgery did not increase the mortality risk, worsen perioperative outcomes, or compromise safety, even in the elderly or patients with more comorbidities [6, 12, 26–28]. Furthermore, the Maze procedure may maintain sinus rhythm and reduce late mortality. According to the current consensus, concomitant surgical ablation with major cardiac surgery is reasonable in patients with symptomatic AF [25]. The effectiveness of the Maze procedure for treating AF is well established, and this procedure is currently the gold standard of surgical ablation [6]. These factors along with the increased incidence of AF may have contributed to the increased use of the Maze procedure in Taiwan, similar to worldwide trends [1, 29]. The slight trend of Maze procedures toward lower-volume hospitals observed in Taiwan may be due to the increased willingness of low-volume physicians to perform concurrent surgical ablation of AF based on the clinical benefits and advanced modern practices described above.

### Relationship between surgical volumes and short- and long-term outcomes

Our study demonstrates the inverse relationship between surgical volumes and both short- and long-term outcomes. Lower-volume hospitals encountered a higher risk of postoperative complications, including postcardiotomy shock and re-exploration, and in-hospital mortality. The more complex comorbidities and complicated cardiac lesions may have impacted postoperative outcomes [20, 30], and these cases should be performed by highly experienced surgeons [20]. In addition, the refinement of operative techniques/equipment and perioperative care are crucial to improving clinical outcomes [31]. Nevertheless, the lower-volume hospitals in our study were more likely to be non-center (regional or district) hospitals where the perioperative healthcare capacity may not be sufficient for patients with more complicated cardiac defects and physical frailty.

Several previous reports demonstrated a positive hospital volume-outcome relationship. However, these results were mainly related to in-hospital (short-term) outcomes, especially mortality [32]. Our study revealed more favorable long-term outcomes for higher–volume hospitals compared with outcomes for lower-volume hospitals, establishing the positive volume-outcome relationship. Lower-volume hospitals tended to have a greater adjusted risk of all-cause mortality and the mortality after discharge. Despite the popularization of the concomitant Maze procedure and advanced procedure techniques and equipment in Taiwan, the positive volume-outcome relationship has not been diminished or eliminated in the past decade as expected. Surgeons’ experience and skill are constantly thought to affect the outcomes of the concomitant Maze procedure [10, 12, 13].

Our study demonstrated that the rate of AF patients receiving concomitant surgical ablation when undergoing major cardiac surgery was higher in the highest-volume hospitals (47.4%) than in the lowest-volume hospitals (18.8%). Niv et al. demonstrated that limited surgeon experience of surgical ablation and a higher EuroSCORE predicted the lack of Maze procedure performance with concurrent valve surgery [10]. They also reported that surgeons were eight times less likely to perform Maze procedures if they experienced fewer than 50 cases [13]. Thus, to achieve optimal outcomes, further education and performance training are necessary. In addition to physician skill/experience, experienced multidisciplinary teams and comprehensive care processes influence clinical outcomes [33]. An early study also indicated that patients should be referred to high-volume hospitals to reduce avoidable mortality [34]. Thus, we suggest that patients with more comorbidities or requiring complicated cardiac surgery should undergo procedures in experienced higher-volume hospitals where adequate healthcare can be provided.

Although the relationships between procedural volume and outcomes for different cardiac surgeries have been demonstrated [14–16, 20], the volume–outcome relationship for the Maze procedure has not been well established. To the best of our knowledge, the present study is one of a limited number of reports to analyze the volume–outcome relationship for the Maze procedure. Using the universal coverage Taiwan NHI system database, we analyzed a large-scale nationwide population. In addition, we conducted statistical adjustments to reduce the confounding variables. With refined methodology, the results of this analysis may influence healthcare policy and clinical practice [35].

### Limitations

There are several limitations to our study. Follow-up electrocardiograms could not be retrieved from the database; thus, we could not evaluate the outcomes of sinus rhythm restoration. Nevertheless, we demonstrated the higher rate of absence of anticoagulant uses in highest-volume hospitals during follow-up though it may not directly indicate sinus rhythm restoration. In addition, some perioperative data could not be obtained, especially details about Maze procedure, such as Maze lesion set and energy source. However, since we analyzed data from the NHIRD with a large-scale population and reliable Death Registry database interpretation (providing the date and cause of deaths), this study reflects the crucial real-world long-term outcomes. We adopted the hospital perspective rather than surgeons to calculate the volume effect since major cardiac surgeries with concomitant Maze are much more complicated, needing the support of associated specialty teams. Furthermore, surgeons may be transferred to different hospitals and their privacy information, including identities and surgery details, could not be acquired. Thus, calculating volume effects based on surgeons would be inappropriate and inaccurate.

## Conclusion

In this population-based cohort study, we establish a positive hospital volume-outcome relationship for the concomitant Maze procedure, including both in-hospital and late follow-up mortality. The effects of hospital volume on concomitant Maze procedure outcomes may be attributed to physician skill/experience, experienced multidisciplinary teams, and comprehensive care processes. Moreover, patients treated in lower volume hospitals generally had more comorbidities. Consequently, we suggest referring frail patients or patients requiring complicated cardiac surgeries to high-volume hospitals to improve clinical outcomes.

## Supplementary Information

**Additional file 1.**  Supplementary Tables. **Supplemental Table 1**. ICD codes used for analysis in the current study. **Supplemental Table 2**. Rate of concomitant surgical ablation for AF patients undergoing major cardiac surgery in each quartile. **Supplemental Table 3**. Rate of concomitant surgical ablation for AF patients undergoing major cardiac surgery according to cardiac procedure. **Supplemental Table 4**. Calculation of Schoenfeld partial residuals for proportional hazard assumptions for each outcome. **Supplemental Table 5**. A mixed-effects model incorporating cluster-specific random effects was used as a sensitivity test for late outcomes.

## Data Availability

All data generated or analyzed during this study are included in this published article and its supplementary information files.
