# Supplementary material for: Surgical volume and outcomes of surgical ablation for atrial fibrillation: a nationwide population-based cohort study
Source: BMC Cardiovasc Disord. 2023 Feb 11;23:84. doi: 10.1186/s12872-023-03101-5 (PMC9922454; doi:10.1186/s12872-023-03101-5)
Supplement: Supplementary file 1 — Additional file 1. Supplementary Tables. Supplemental Table 1. ICD codes used for analysis in the current study. Supplemental Table 2. Rate of concomitant surgical ablation for AF patients undergoing major cardiac surgery in each quartile. Supplemental Table 3. Rate of concomitant surgical ablation for AF patients undergoing major cardiac surgery according to cardiac procedure. Supplemental Table 4. Calculation of Schoenfeld partial residuals for proportional hazard assumptions for each outcome. Supplemental Table 5. A mixed-effects model incorporating cluster-specific random effects was used as a sensitivity test for late outcomes. [file 12872_2023_3101_MOESM1_ESM.docx]

**Supplemental Table 1:** ICD codes used for analysis in the current study.

| **Variable** | **Code** |
| --- | --- |
| Surgical Ablation | ICD-9-OP codes: 37.33 |
|  | ICD-10-PCS codes : 02560*, 02570*, 025S0*, 025T0*, 02T80* |
| Diabetes mellitus [DM] | ICD-9-CM: 250* |
|  | ICD-10-CM: E08-E13 |
| Hyperlipidemia [HL] | ICD-9-CM: 272* |
|  | ICD-10-CM: E77, E780, E781, E782, E783, E784, E785, E786, E881, E753, E755, E882, E756, E789, E7521, E7522, E7524, E7130, E7879, E7881, E7889, E8889, E7870 |
| Hypertension [HTN] | ICD-9-CM: 401*, 402*, 403*, 404*, 405* |
|  | ICD-10-CM: I10-I15, N262 |
| Chronic kidney disease [CKD] | ICD-9-CM: |
|  | 580~589, 403~404 |
|  | 016.0, 095.4, 236.9 250.4, 274.1, 442.1,, 447.3,,440.1, 572.4, 642.1, 646.2 753.1, |
|  | 283.11, 403.01,and 404.02, 446.21 |
|  | ICD-10-CM: |
|  | A1811, D593, E102, E112, E132, I12, I13, K767, M103, M310, N00, N01, N02, N03, N04, N05, N06, N07, N08, N14, N150, N158, N159, N16, N171, N172, N18, N19, N200, N25, N261, N269, N27, Q61 |
| Myocardial infarction [MI] | ICD-9-CM: 410*, 412* |
|  | ICD-10-CM: I21-I22 |
| Heart failure [HF] | ICD-9-CM: 428* |
|  | ICD-10-CM: I50 |
| Stroke | ICD-9-CM: 430*~437* |
|  | ICD-10-CM: I60-I62, I66, I65.1, I65.0, I65.8, I65.9, I63.6, I63.8, I63.9, G45.0, G45.8, G45.1, G45.2, G46.0, G46.1, G46.2, G45.9, G45.4, G46.3, G46.4, G46.5, G46.6, G46.7, G46.8, I67.0, I67.1, I67.2, I67.4, I67.5, I67.6, I67.7, I67.9, I68.0, I68.2, I68.8 |
| Malignant dysrhythmia [MD] | ICD-9-CM: 427.1, 427.4x |
|  | ICD-10-CM: I49.01, I47.2 |
| GI bleeding [GIBD] | ICD-9-CM: 530.21, 530.7, 530.82, 531*~534*,535*, 537.83, 537.84, 578* |
|  | CD-10-CM: K22.6, K25.0, K26.0, K27.0, K28.0, K62.5, K29.01, K29.31, K29.61, K29.21, K29.31, K29.71, K29.81, K52.81, K31.811, K31.82, K57.11, K57.01, K57.31, K57.21, K55.21, K92.0, K31.82, K56.60, K27.1, K28.1,, K29.41, K29.61, K29.91,K57.13, K57.91, K57.33,K92.1, K56.60, K26.1, K27.2, K28.2,, K29.51, K52.81, K57.81,K92.2, K25.1, K26.2, K27.3, K28.3,, K57.93, K25.2, K26.3, K27.4, K28.4, K25.3, K26.4, K27.5, K28.5, K25.4, K26.5, K27.6, K28.6, K25.5, K26.6, K27.7, K28.7, K25.6, K26.7, K27.9, K28.9, K25.7, K26.9, K25.9 |
| Rheumatic heart disease [RHD] | ICD-9-CM: 394.0, 394.1, 394.2, 395*, 398.9* |
|  | ICD-10-CM: I05-I09 |
| History of PCI | 33076*, 33077*, 33078*, |
| History of EP ablation | 33091A, 33091B, 68050B |
| CABG | 68023, 68024, 68025, 68053, 68054, 68055 |
| AV mechanical | AV replacement with mechanical valve: |
|  | ICD-9 OP: 3522 |
|  | ICD-10 OP: 02RF0J* |
| AV tissue | AV replacement with bioprosthetic valve |
|  | ICD-9 OP: 3521 |
|  | ICD-10 OP: 02RF08*, 02RF0K* |
| MV repair | MV_repair |
|  | ICD-9 OP: 3512 |
|  | ICD-10 OP: 02QG0*, 02UG0JZ |
| MV replacement mechanical | MV replacement with mechanical valve |
|  | ICD-9 OP: 3524 |
|  | ICD-10 OP: 02RG0J* |
| MV replacement tissue | MV replacement with bioprosthetic valve |
|  | ICD-9 OP: 3523 |
|  | ICD-10 OP: 02RG0K*, 02RG08* |
| TV repair | TV repair |
|  | ICD-9 OP: 3514 |
|  | ICD-10 OP: 02QJ0*, 02UJ0JZ |
| TV replacement | TV replacement with mechanical valve |
|  | ICD-9 OP: 3528 |
|  | ICD-10 OP: 02RJ0J* |
| Aortic surgery | 69024, 69035, 69036, 69037, 68043 |

**Supplemental Table 2:** Rate of concomitant surgical ablation for AF patients undergoing major cardiac surgery in each quartile.

|  | Number of patients undergoing concomitant surgical ablation | Number of patients undergoing cardiac surgical procedures | Rate of concomitant surgical ablation for AF patients (%) |
| --- | --- | --- | --- |
| Quartile 1 | 678 | 3601 | 18.8 |
| Quartile 2 | 692 | 1681 | 41.2 |
| Quartile 3 | 768 | 2414 | 31.8 |
| Quartile 4 | 528 | 1114 | 47.4 |
| Total | 2666 | 8810 | 30.3 |

AF, atrial fibrillation; Quartile 1, lowest; Quartile 4, highest.

**Supplemental Table 3:** Rate of concomitant surgical ablation for AF patients undergoing major cardiac surgery according to cardiac procedure.

|  | Overall cohort | Q1 | Q2 | Q3 | Q4 | *P*-value  (chi-square for trend) |
| --- | --- | --- | --- | --- | --- | --- |
| CABG | 13.5 | 9.0 | 16.7 | 11.0 | 25.5 | <0.001 |
| Mechanical AVR | 24.4 | 19.2 | 33.6 | 18.7 | 52.1 | <0.001 |
| Tissue AVR | 29.4 | 19.3 | 44.0 | 26.1 | 33.6 | 0.015 |
| MV repair | 51.8 | 30.9 | 65.8 | 59.6 | 59.3 | <0.001 |
| Mechanical MVR | 39.1 | 27.5 | 57.8 | 43.8 | 66.0 | <0.001 |
| Tissue MVR | 48.2 | 34.4 | 62.3 | 38.6 | 63.0 | <0.001 |
| TV repair | 44.9 | 33.0 | 62.1 | 44.5 | 63.9 | <0.001 |
| TVR | 27.1 | 16.4 | 55.8 | 17.3 | 27.8 | 0.91 |
| Aorta surgery | 11.7 | 15.3 | 7.0 | 10.5 | 12.9 | 0.51 |

Quartile 1, lowest; Quartile 4, highest; AF, atrial ﬁbrillation; CABG, coronary artery bypass graft; AVR, aortic valve replacement; MV, mitral valve; MVR, mitral valve replacement; TV, tricuspid valve; TVR, tricuspid valve replacement. Data are presented as percentages (%).

**Supplemental Table 4**: Calculation of Schoenfeld partial residuals for proportional hazard assumptions for each outcome.

|  | Proportional hazards assumption (*P*-value) |
| --- | --- |
| **All-cause mortality** |  |
| Q2 vs. Q1 | 0.63 |
| Q3 vs. Q1 | 0.94 |
| Q4 vs. Q1 | 0.052 |
| **All-cause mortality after discharge** |  |
| Q2 vs. Q1 | 0.27 |
| Q3 vs. Q1 | 0.93 |
| Q4 vs. Q1 | 0.18 |
| **Readmission (CV-related causes]** |  |
| Q2 vs. Q1 | 0.73 |
| Q3 vs. Q1 | 0.75 |
| Q4 vs. Q1 | 0.72 |
| **Readmission (all cause, within 30 days)** |  |
| Q2 vs. Q1 | 0.90 |
| Q3 vs. Q1 | 0.39 |
| Q4 vs. Q1 | 0.38 |
| **MACE** |  |
| Q2 vs. Q1 | 0.29 |
| Q3 vs. Q1 | 0.79 |
| Q4 vs. Q1 | 0.39 |

Quartile 1, lowest; Quartile 4, highest; MACE, major adverse cardiovascular events.

**Supplemental Table 5:** A mixed-effects model incorporating cluster-specific random effects was used as a sensitivity test for late outcomes.

| Event type | Group | HR (95%CI) | P value |
| --- | --- | --- | --- |
| **All-cause mortality** | Q2 vs. Q1 | 0.98 (0.79–1.22) | 0.89 |
|  | Q3 vs. Q1 | 0.72 (0.56–0.91) | 0.007 |
|  | Q4 vs. Q1 | 0.52 (0.39–0.70) | <0.001 |
| **All-cause mortality after discharge** | Q2 vs. Q1 | 1.08 (0.84–1.37) | 0.55 |
|  | Q3 vs. Q1 | 0.71 (0.54–0.93) | 0.012 |
|  | Q4 vs. Q1 | 0.60 (0.44–0.80) | <0.001 |
| **Readmission (CV-related causes)** | Q2 vs. Q1 | 0.95 (0.79–1.14) | 0.56 |
|  | Q3 vs. Q1 | 0.77 (0.63–0.95) | 0.012 |
|  | Q4 vs. Q1 | 0.88 (0.68–1.14) | 0.32 |
| **Readmission (all cause, within 30 days)** | Q2 vs. Q1 | 0.88 (0.63–1.24) | 0.47 |
|  | Q3 vs. Q1 | 0.66 (0.46–0.96) | 0.027 |
|  | Q4 vs. Q1 | 0.81 (0.56–1.15) | 0.24 |
| **MACE** | Q2 vs. Q1 | 1.42 (0.95–2.14) | 0.091 |
|  | Q3 vs. Q1 | 1.13 (0.75–1.71) | 0.55 |
|  | Q4 vs. Q1 | 1.07 (0.69–1.67) | 0.75 |

Quartile 1, lowest; Quartile 4, highest; MACE, major adverse cardiovascular events.
